# Microbial adaptation to the healthy and inflamed gut environments

**DOI:** 10.1080/19490976.2020.1857505

**Published:** 2020-12-17

**Authors:** Yijie Guo, Sho Kitamoto, Nobuhiko Kamada

**Affiliations:** a Division of Gastroenterology and Hepatology, Department of Internal Medicine, University of Michigan, Ann Arbor, MI, USA; b Department of Pathogenic Biology and Immunology, School of Basic Medical Sciences, Xi’an Jiaotong University, Xi’an, Shaanxi, China

**Keywords:** Gut microbiota, commensal bacteria, pathogenic bacteria, intestinal inflammation

## Abstract

There are 100 trillion diverse bacterial residents in the mammalian gut. Commensal bacterial species/strains cooperate and compete with each other to establish a well-balanced community, crucial for the maintenance of host health. Pathogenic bacteria hijack cooperative mechanisms or use strategies to evade competitive mechanisms to establish infection. Moreover, pathogenic bacteria cause marked environmental changes in the gut, such as the induction of inflammation, which fosters the selective growth of pathogens. In this review, we summarize the latest findings concerning the mechanisms by which commensal bacterial species/strains colonize the gut through cooperative or competitive behaviors. We also review the mechanisms by which pathogenic bacteria adapt to the inflamed gut and thrive at the expense of commensal bacteria. The understanding of bacterial adaptation to the healthy and the inflamed gut may provide new bacteria-targeted therapeutic approaches that selectively promote the expansion of beneficial commensal bacteria or limit the growth of pathogenic bacteria.

## 1. Introduction

The gut microbiota is a vast community of commensal microorganisms that reside in the gastrointestinal (GI) tract. These microorganisms are from multiple kingdoms, including bacteria, fungi, and viruses, each comprising numerous different members of microbes, with high diversity. For example, the kingdom Bacteria within the gut microbiota comprises over 1,000 different bacterial species. Each species or strain of gut commensal bacteria performs unique biological functions, and therefore, their balance is vital for the maintenance of GI homeostasis. The perturbation of this balance – so-called gut dysbiosis – triggers or exacerbates various GI diseases, such as inflammatory bowel disease (IBD).^1^ Each bacterial species or strain uses a wide variety of mechanisms to adapt to the gut microenvironment and to stabilize its colonization. Importantly, the colonization of each bacterium is influenced by the presence of other bacteria, through both cooperative and competitive behaviors. In this review, we will discuss the mechanisms by which gut bacteria adapt to the gut microenvironment, both in the steady state and in disease.

## 2. Bacterial adaptation in the steady-state gut

**Figure 1.**
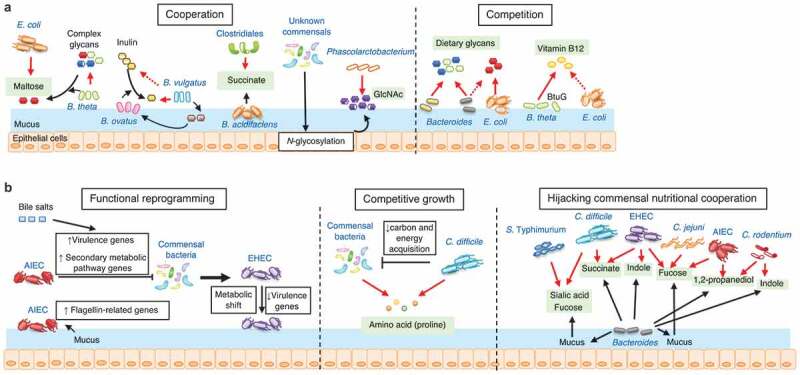
Mechanisms for the adaptation of commensal and pathogenic bacteria in the steady-state gut

Commensal bacteria have evolved various strategies to adapt to the gut environment, mainly through the acquisition of available nutrients. In this section, we will summarize the mechanisms by which commensal bacteria sense and adapt to the gut environment, through their metabolic landscape. Also, metabolic cooperation and competition between bacterial strains will be discussed. Some examples of the strategies used by pathogenic bacteria to establish infection at the expense of competing commensal bacteria will be described Figure 1.

(A) Commensal bacterial species or strains cooperate or compete with other bacteria to establish the colonization of each bacterial strain. Cooperation: Gut resident *B. thetaiotaomicron* (*B. theta*) liberates maltose from dietary glycans, which, in turn, promotes the growth of *E. coli. B. ovatus* degrades dietary inulin and generates inulin derivatives. *B. vulgatus*, which fails to break down inulin, can be fed by the inulin derivatives. The expanded population of *B. vulgatus* supports the growth of *B. ovatus*. Clostridiales owe the successful establishment of their colonization to succinate produced by other bacteria, such as *B. acidifaciens*. The colonization by gut commensals increases the *N*-glycosylation of host epithelial cell or mucosal proteins. *Phascolarctobacterium* uses *N*-acetylglucosamine (GlcNAc) released from host proteins. Competition: *Bacteroides* species compete with each other for dietary glycans; whereas, they do not compete for glycans with metabolically unrelated species, such as *E. coli*. In contrast, *B. thetaiotaomicron* uses the lipoproten BtuG to compete with *E. coli* for vitamin B12. (B) Functional reprogramming: When exposed to the gut microenvironment, pathogenic bacteria reprogram their functions to establish a stable colonization. Adherent–invasive *E. coli* (AIEC) modulate the expression of virulence and metabolic pathway genes through the sensing of gut metabolites (e.g., bile salts) or mucus. Enterohemorrhagic *E. coli* (EHEC) alters the expression of virulence or metabolic pathway genes in the presence of commensal microbiota, thereby changing its behavior in the gut. Competitive growth: *C. difficile* competes for proline with commensal bacteria. *C. difficile* infection results in the reduction of carbon and energy acquisition genes in commensals, allowing the pathogen to occupy the nutrient niche. Hijacking commensal nutritional cooperation: Several pathogenic bacteria harbor strategies to use the products generated by commensal bacteria for their growth. The gut colonization of *Bacteroides* species results in the generation of metabolic by-products, such as sialic acid, fucose, succinate, indole, and 1,2-propanediol. These metabolites are often used for the cooperative growth of other commensal bacteria. Pathogens can hijack nutritional cooperation systems and use these metabolites for their own growth to establish infection.

### 2.1. Environmental sensing by commensal bacteria

Nutritional and geographical adaptation are two of the most critical mechanisms that commensal bacteria use to colonize and grow in the gut. In the gut lumen, plant-derived indigestible complex polysaccharides, which are provided through the food consumed by the host, are the major nutrient source for resident bacteria. On the other hand, in close proximity to the mucosa, host-derived polysaccharides, such as mucus glycans, provide an alternate nutrient source. The ability to digest plant- or host-derived polysaccharides is a key factor contributing to the spatial adaptation to the gut environment by commensal bacteria. For example, commensal *Bacteroides* species possess unique gene clusters called polysaccharide utilization loci (PULs).^2^ PULs contain various genes associated with the use of a wide variety of plant- or host-derived complex glycans, such as enzymes that breakdown complex glycans, as well as transporters for generated simple sugars.^3,4^ PULs determine the niche adaptation of *Bacteroides* species in the gut. Based on the available polysaccharides in their surroundings, *Bacteroides* species selectively express corresponding PULs to adapt to the environmental niche immediately.^3^ In commensal *B. fragilis*, a unique class of PULs – commensal colonization factors (CCF) – is critical for the adaptation to the gut.^5^ The *ccf* genes are upregulated at the colonic surface, thereby enabling *B. fragilis* to penetrate the colonic mucus and stably localize at the bottom of the crypts.^5^ The expression of *ccf* genes in *B. fragilis* regulates the biosynthesis of capsular polysaccharides (PS).^6^ CCF-dependent regulation of PS induces the development of PS-specific IgA responses by the host.^6^ IgA binding to *B. fragilis* capsular PS increases the adherence to intestinal epithelial cells, thereby enhancing colonization stability at the mucosal niche.^6^ In addition to CCF-mediated transcriptional regulation, the synthesis of capsular PS in *B. fragilis* is influenced by PS locus promoter orientation.^7^ PS locus promoters contain DNA segments that undergo reversible inversions.^8^ PS locus promoters are therefore placed in the correct (i.e., ON) or incorrect (i.e., OFF) orientation that regulates the transcription of PS biosynthesis genes.^7^ Although the factors driving the promoter orientations of *B. fragilis* in the gut are incompletely understood, the environmental factors, such as the presence of complex microbiota, influence the promoter orientations.^7^ Capsular PS enhance the stability of the bacterium at the mucosal niche,^6^ and also elicit host immune responses, which may in turn influence bacterial colonization.^9–11^ Therefore, PS promoter orientations may be important for the adaptation of bacteria – commensals and potential pathogens – in the gut environment. In this context, a significantly higher proportion of gut *B. fragilis* strains had the PSA promoter oriented OFF in individuals with IBD compared to healthy individuals.^12^ Thus, the sensing of the surrounding environment (nutritional or spatial) triggers the expression of appropriate genes and facilitates the adaptation of commensal bacteria to the specific gut environment. In-depth studies of the spatial adaptation mechanisms deployed by commensal bacteria are ongoing. Using hybrid selection RNA sequencing, Donaldson *et al*. discriminated the transcriptomic profile of the commensal *B. fragilis* in the mucosal niche from that in the gut lumen.^13^ *B. fragilis* colonized in the mucosal niche is significantly more metabolically active than *B. fragilis* localized to the lumen, as evidenced by the upregulation of numerous genes for protein synthesis, including genes that likely encode acetylglucosamine-6-sulfatase (BF3086) and cyclomaltodextrinase (BF3134), which are important for mucin glycan foraging.^13^ In the colonic mucus of gnotobiotic mono-colonized mice, mutant *B. fragilis* (∆BF3086 and ∆BF3134) showed significantly lower levels than wildtype bacteria. Therefore, *B. fragilis* deploys a specific genetic program at distinct sites within the gut.^13^

### 2.2. Metabolic cross talk between commensal bacteria

#### 2.2.1. Cooperation (cross feeding)

Different species or strains of commensal bacteria in the gut harbor distinct nutritional preferences that are used to adapt to the gut environment. The specific nutritional habit is, therefore, a vital determinant of the balance between each species or strain within the gut microbiota. Some commensal bacteria develop a mutualistic relationship with other bacteria and cooperatively establish the colonization of each bacterial strain Figure 1a. This phenomenon is termed cross feeding.^14,15^ For example, *Escherichia coli* and *Bacteroides thetaiotaomicron* both colonize the steady-state gut, but each has a different capability in the use of glycans for their growth. *E. coli* lacks glycoside hydrolase activity for dietary glycan, whereas *B. thetaiotaomicron* is a glycophile, and thus, is able to break down a broad array of dietary glycans. In *E. coli* mono-colonized mice, the expression of genes for maltose utilization is increased when the mice are co-colonized with *B. thetaiotaomicron*. This observation indicates that *E. coli* can be fed by *B. thetaiotaomicron*, which liberates maltose from dietary glycans.^16^ Tuncil *et al*. found that human gut *Bacteroides* species, such as the closely related species *B. ovatus* and *B. thetaiotaomicron*, have cognate polysaccharide utilization loci for degrading a number of dietary glycans. However, they do not simultaneously use all of these glycans. Each species displays variable and sometimes opposite rank orders for some glycans, thereby maintaining their stable coexistence in a competitive environment.^17^ Rakoff-Nahoum *et al*. discovered a dedicated cross-feeding enzyme system in the gut symbiont *B. ovatus*, which extracellularly digests and liberates considerable amounts of inulin breakdown products to benefit other species that cannot use inulin, such as *B. vulgatus*. In return, *B. vulgatus* can benefit *B. ovatus* by detoxifying inhibitory substances and producing growth-promoting factors, thereby supporting the growth of *B. ovatus*.^18^ Moreover, some commensal bacterial strains rely entirely on the metabolic functions of cooperative partners to establish their colonization in the gut. Kim *et al*. showed that Clostridiales bacteria fail to colonize germ-free (GF) mice, whereas Clostridiales successfully establish colonization if the GF mice are pre-colonized with some commensal bacteria, such as *B. acidifaciens*.^19^ The pre-colonized *B. acidifaciens* produces succinate, which enhances the growth of Clostridiales.^19^ This metabolic cross talk between *B. acidifaciens* and Clostridiales is essential for the maturation of the gut microbiota from neonate to adult. Likewise, the colonization of the gut microbiota alters the *N*-glycosylation of the host mucus or epithelial proteins, which, in turn, foster the expansion of *Phascolarctobacterium*, which can use host-derived glycans as nutrients.^20^

#### 2.2.2. Competition

In addition to cooperative behaviors, bacterial species and strains in the gut can also compete with each other to maintain abundance at the expense of other bacteria. Nutritional (metabolic) competition is one of the major regulatory forces of the gut bacterial community Figure 1a. For example, many members of the genus *Bacteroides* share the same mechanism for digesting multiple diet- and host-derived glycans, whereby they compete with each other for nutrients in the gut lumen.^21^ Likewise, Lee *et al*. reported that *Bacteroides* colonize the gut in a species-specific and saturable manner. Single *Bacteroides* species mono-associated mice are resistant to colonization by the same, but not different, species (e.g., *E. coli*).^5^ CCF is a determinant for the interspecies niche competition, as CCF deletion in *B. fragilis* results in colonization defects and reduced horizontal transmission. In addition to carbohydrates, other nutrients, such as vitamins, are also essential for commensal fitness. *B. thetaiotaomicron*, unlike *E. coli*, is replete with conserved genes encoding proteins for three functional vitamin B12 acquisition systems.^22,23^ In particular, BtuG, a surface-exposed lipoprotein essential for efficient vitamin B12 transport in *B. thetaiotaomicron*, binds vitamin B12 with femtomolar affinity and removes vitamin B12 from the host’s own vitamin B12 collecting protein, thus enhancing its fitness. When *B. thetaiotaomicron*, with and without BtuG, is placed in a gnotobiotic mouse model, the *B. thetaiotaomicron* lacking functional BtuG is outcompeted by the bacteria with functional BtuG, suggesting that BtuG-mediated capture of vitamin B12 is a crucial factor for the fitness of this strain.

### 2.3. Establishing infection by pathogenic bacteria

Similar adaptation mechanisms are used by pathogenic bacteria to establish an infection in the gut Figure 1b. Adherent–invasive *E. coli* (AIEC) exhibits the expression of genes related to flagella formation when it senses the gut environment, such as the presence of bile salts and mucus.^24^ The expression of flagellin-related genes, in turn, promotes the penetration of AIEC into the mucus layer and transit to the epithelial surface.^24^ As flagellin is a critical factor for AIEC pathogenesis,^25^ this is an essential adaptation mechanism that enables AIEC to establish disease-causing colonization. Bile salts can also modulate AIEC expression of other virulence genes and secondary metabolic pathway genes, conferring the fitness advantage to AIEC over competing indigenous bacteria.^26^ Enterohemorrhagic *E. coli* (EHEC) reprograms its gene transcription when it is cultured in cecal content isolated from human microbiota–associated rats.^27^ EHEC shifts its metabolism from glycolytic to anaplerotic.^27^ The *ex vivo* gut environment inhibits the expression of various virulence genes involved in attaching and effacing lesion formation by EHEC.^27^ The downregulation of virulence factors may be necessary to promote faster growth of the pathogen population, as virulence expression is costly.^28^ EHEC may occupy the spatial or nutritional niches, forcing out commensal competitors by growing faster. On achieving sufficient robustness in the gut, they may turn on the virulence to establish infection. Moreover, *Clostridioides difficile* uses amino acids, particularly proline, available in the gut lumen to grow and subsequently establish infection.^29^ In the presence of commensal bacteria, especially metabolically associated Clostridia, *C. difficile* competes for proline, which limits its growth.^30^ The absence of competitors, as occurs in germ-free mice, in patients with diarrhea, and with antibiotic treatment, enhances the availability of free proline in the gut lumen, thereby fostering the growth of *C. difficile*.^29^ Of note, once *C. difficile* has established infection, it induces a shift in the transcriptional activities of the commensal microbiota, particularly a minority subset of species.^31^ As a result, genes associated with carbon and energy acquisition are greatly reduced in the gut microbiota, which, in turn, allows *C. difficile* to occupy a nutrient niche and sustain infection.^31^

In addition to the nutritional competition between pathogens and commensals, pathogens often use nutrient sources offered by commensal bacteria to help them adapt to the gut environment (i.e., nutritional cooperation). Ng *et al*. found that *Salmonella enterica* serovar Typhimurium (*S. enterica* ser. Typhimurium) and *C. difficile* use sialic acid and fucose, liberated from the host glycans by symbiotic *Bacteroides*, as carbohydrate sources.^32^ Likewise, EHEC and *C. difficile* use succinate generated by the *B. thetaiotaomicron*–colonized murine gut.^33^ *Citrobacter rodentium*, a mouse pathogen that models EHEC and enteropathogenic *E. coli* (EPEC) in humans, uses 1,2-propanediol produced by *B. thetaiotaomicron* and converts it to propionate, which, in turn, promotes the expression of LEE1 virulence genes.^34^ *Campylobacter jejuni*, a non-saccharolytic bacterium, can catabolize l-fucose released by commensal *B. vulgatus* to scavenge enough carbohydrate nutrient for efficient proliferation and colonization.^35^ EHEC senses the fucose released from the mucus by *B. thetaiotaomicron*, and modulates its metabolism by way of the two-component signal transduction system FusKR, optimizing growth and decreasing competition with commensal *E. coli* for carbon sources.^36^ Fucose also promotes AIEC proliferation through propanediol dehydratase (PduC), suggesting a capacity for intestinal adaptation by catabolizing nutrients liberated from the host glycans.^37^ Furthermore, EHEC and *C. rodentium* use indole generated by commensal *B. thetaiotaomicron* through the histidine kinase CpxA to downregulate LEE virulence gene expression during colonization in the colonic lumen, while the repression is lifted at the mucosa due to lower indole concentrations as the result of host cell absorption.^38,39^

## 3. Bacterial adaptation in the inflamed gut

**Figure 2.**
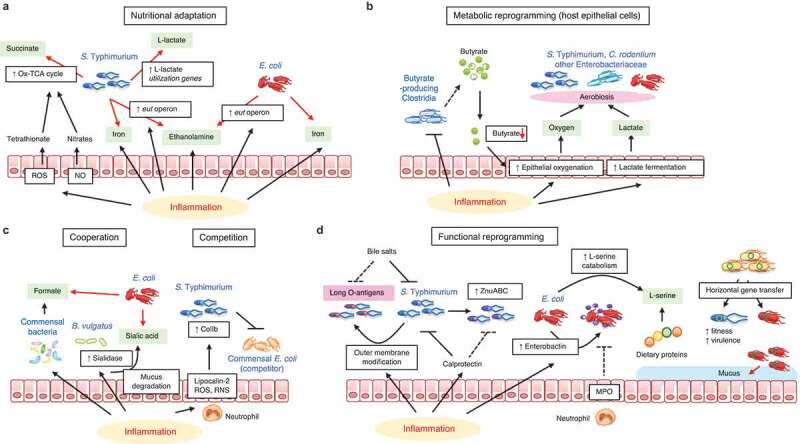
Pathogenic bacteria adaptations in the inflamed gut

Pathogens and pathobionts are enriched with genes linked to flagella, secretion systems, adhesins, and proteins involved in biofilm formation. All facilitate the invasion of the mucus barrier and adherence to the intestinal epithelium, which have been regarded as key determinants of the inflammatory response.^40^ Gut inflammation caused by pathogens significantly alters the gut microenvironment, which, in turn, impacts the fitness of bacteria in the gastrointestinal tract, thereby shaping the structure of the resident microbial community. For example, inflammation results in the elevation of certain nutrients that selectively promote the growth of pathogenic bacteria. Also, metabolic by-products released by inflamed host cells support the proliferation of some, most likely pathogenic, bacteria. Moreover, inflammation elicits metabolic reprogramming (e.g., transcriptional regulation, horizontal gene transfer) and, as a result, pathogens acquire functions that enable them to adapt to the inflammatory milieu. In this section, we will discuss microbial adaptation mechanisms in the inflamed gut Figure 2.

(A) Nutritional adaptation: Intestinal inflammation induces reactive oxygen species (ROS) and nitric oxide (NO), which lead to the generation of tetrathionate and nitrates, respectively. These electron acceptors induce the oxidative TCA cycle (Ox-TCA cycle) in *S. enterica* ser. Typhimurium, conferring the growth advantage to this pathogen over commensals. Inflammation likewise induces the transcription of the *eut* operon in *S. enterica* ser. Typhimurium and pathogenic *E. coli*, enabling the pathogens to grow on ethanolamine, which is induced during inflammation. (B) Metabolic reprogramming (host epithelial cells): Intestinal inflammation shifts the metabolism of intestinal epithelial cells (IECs). In the inflamed gut, the reduction of butyrate-producing Clostridia and subsequent decrease in luminal butyrate levels lead to epithelial oxygenation and lactate fermentation. The resulting elevation of oxygen and lactate, produced by IECs, promotes the aerobiotic growth of *Enterobacteriaceae* pathogens, such as *S. enterica* ser. Typhimurium and *C. rodentium*. (C) Cooperation or Competition: Intestinal inflammation promotes gut dysbiosis. The dysbiotic microbiota alters its metabolic function, thereby generating formate. Likewise, inflammation changes sialidase activity in *B. vulgatus*, increasing sialic acid release from mucin glycans. Upregulated formate and sialic acid, in turn, support the growth of *E. coli* in the inflamed gut. *S. enterica* ser. Typhimurium ColIb production is induced by inflammatory mediators, such as lipocalin-2, ROS, and reactive nitrogen species (RNS). ColIb limits the growth of commensal *E. coli* and promotes the competitive fitness of *S. enterica* ser. Typhimurium. (D) Functional reprogramming: Intestinal inflammation induces antimicrobial responses that limit the growth of the resident bacteria. Pathogenic bacteria have evolved strategies to evade such antimicrobial responses through functional reprogramming. *S. enterica* ser. Typhimurium modifies its outer membranes in the inflamed gut. The very long O-antigens chains of *S. enterica* ser. Typhimurium inhibit the bactericidal effect of bile salts. Also, *S. enterica* ser. Typhimurium expresses the high-affinity zinc transporter ZnuABC to resist the inflammation-induced calprotectin-mediated zinc chelation. Pathogenic *E. coli* secretes enterobactin to dampen the activity of myeloperoxidase (MPO), and thus evades immune-mediated killing. Pathogenic *E. coli* increases transcription of l-serine catabolism genes in the inflamed gut, thus gaining a growth advantage over commensal competitors by using dietary protein–derived l-serine. Also, some pathogenic bacteria acquire novel functions through horizontal gene transfer to adapt better to the surrounding environment (e.g., growth on host mucus glycans).

### 3.1. Using inflammatory niches for the growth of pathogens

#### 3.1.1. Pathogens survive and proliferate in the inflamed gut

Enteropathogenic bacteria are prone to survive and proliferate in the inflamed gut. As described in Figure 2a, during *Salmonella*-induced gastroenteritis, mucosal inflammation creates a niche that favors the expansion of the pathogen population over the commensal bacteria. The host inflammatory response in the inflamed gut generates by-products of reactive nitrogen, which can react to form nitrates, which, in an anaerobic environment, are used as respiratory electron acceptors of *S. enterica* ser. Typhimurium to enhance the growth of this pathogen.^41^ Inflammation-derived electron acceptors induce a complete oxidative TCA cycle in *S. enterica* ser. Typhimurium. This oxidative central metabolism enables *S. enterica* ser. Typhimurium to use the microbiota-derived fermentation product succinate as a nutrient to compete with the microbiota.^42^ Iron is an essential trace element that plays a crucial role in the proliferation and virulence of pathogens and pathobionts, as well as in the growth of commensal bacteria.^43,44^ Inflammation causes tissue disruption, which leads to the release of iron. Both gram-negative (e.g., *E. coli, Pseudomonas aeruginosa, Klebsiella pneumoniae*) and gram-positive (e.g., *Staphylococcus aureus*) bacteria acquire iron by synthesizing and secreting diverse siderophores.^45^ Siderophores display a stronger affinity to iron than host iron-binding proteins; hence, bacterial pathogens can acquire iron and gain a growth advantage in the inflamed gut.^46,47^

#### 3.1.2. Pathogens change cellular metabolism in the inflamed gut

The cellular metabolism of intestinal epithelial cells (IECs) is altered during inflammation, which, in turn, affects the growth of certain pathogenic bacteria in the gut Figure 2b. For instance, *Salmonella*-induced colitis drives a depletion of butyrate-producing Clostridia. Due to the lack of butyrate, epithelial oxygenation is increased, causing oxygen to leak from the tissue into the lumen.^48^ The increased oxygen level in the epithelial cells supports the growth of *Salmonella* through high-affinity terminal oxidases in aerobic conditions.^48^ Also, *C. rodentium* can use aerobic respiration in the inflamed gut to gain a growth advantage over commensal bacteria.^49^ Cevallos *et al*. found that dextran sodium sulfate (DSS)-induced colitis increased epithelial oxygenation and the bioavailability of luminal oxygen, thereby driving the expanded growth of *E. coli* among intestinal microorganisms through aerobic respiration.^50^ Gills *et al*. also reported that inflammation alters the metabolism of IECs. In inflammation, IECs display increased lactate fermentation, which leads to the elevation of luminal lactate.^51,52^ *Salmonella* uses lactate as an electron donor in conjunction with oxygen as the terminal electron acceptor to support its colonization and overgrowth in the gut.^51,52^ During *C. rodentium* infection, the IECs shift to cholesterol and carbon metabolism, triggering aerobic glycolysis and activating cholesterol biogenesis and efflux, while dampening central carbon metabolism, in particular, the production of mitochondrial cardiolipins. This coincides with increased mucosal oxygen levels and a reduction in colon-associated anaerobic commensals, thus supporting the expansion of mucosa-associated *Enterobacteriaceae*.^53,54^

#### 3.1.3. Pathogen cooperation and competition in the inflamed gut

Bacterial cooperation or competition with other bacteria are key elements for defining the bacterial fitness in the inflamed gut Figure 2c. DSS-induced intestinal inflammation is often accompanied by an alteration of the gut microbiota (i.e., dysbiosis). Metagenomic sequencing has revealed that bacterial formate oxidation and oxygen respiration are overrepresented metabolic pathways in the dysbiotic microbiome. *E. coli* uses microbiota-derived formate through oxygen respiration to enhance its fitness in the inflamed gut Figure 2c. ^55^ Colicins are bacterial protein toxins that show potent activity against sensitive strains *in vitro. S. enterica* ser. Typhimurium produces colicin Ib (ColIb), a narrow-spectrum protein toxin active against related *Enterobacteriaceae*, such as colicin-sensitive commensal *E. coli*.^56^ The inflammatory environment in the gut provides unique conditions that potentiate the effects of colicins. Neutrophils migrate into the lumen of the inflamed gut and release iron-depleting agents and reactive oxygen and nitrogen species. This significantly induces the genes for ColIb production in *S. enterica* ser. Typhimurium and its corresponding ColIb-surface receptor CirA in commensal *E. coli*. Hence, *S. enterica* ser. Typhimurium produces ColIb, which confers a growth advantage over commensal *E. coli* in the inflamed gut. In contrast, in the absence of gut inflammation, ColIb production does not confer a competitive advantage to *S. enterica* ser. Typhimurium.^56^ Abnormal mucus regulation is a hallmark of intestinal inflammation, linked to the expansion of pathogens. During DSS-induced colitis, the increased release of sialic acids from mucin is caused by the expansion of *B. vulgatus*, which produces a sialidase. Sialic acids are, in turn, incorporated into the bacterial capsule of *Enterobacteriaceae*, such as *E. coli*, thus enhancing fitness Figure 2c ^57,58^

### 3.2. Microbial functional reprogramming of pathogens in the inflamed gut

Inflammation-driven functional reprograming is crucial for allowing pathogens to gain the edge in the inflamed gut. As shown in Figure 2d, *Salmonella*-induced colitis increases the luminal concentration of total bile acid. To resist the elevated bile concentration during colitis, *S. enterica* ser. Typhimurium modifies its outer membranes (i.e., very long O-antigen chains) through regulation of FepE.^59^ This enables *S. enterica* ser. Typhimurium to adapt better to the inflamed gut than commensal bacteria, which are susceptible to bile salts. Also, *S. enterica* ser. Typhimurium expresses genes that are required to tolerate the host antimicrobial responses. *Salmonella*-induced colitis releases calprotectin, an antimicrobial protein secreted from dead neutrophils in the intestinal lumen, which can inhibit bacterial growth by sequestering essential micronutrient metals (e.g., zinc). *S. enterica* ser. Typhimurium expresses a high-affinity zinc transporter (ZnuABC) in the inflamed gut.^60^ ZnuABC gives *S. enterica* ser. Typhimurium a significant fitness advantage over commensal bacteria by overcoming calprotectin-mediated zinc chelation Figure 2d. Likewise, *S. enterica* ser. Typhimurium upregulates the transcription of l-lactate utilization genes to use lactate in the gut lumen as an electron donor.^52^ *S. enterica* ser. Typhimurium and AIEC increase transcription of the *eut* operon to use intestinal ethanolamine, which is secreted from intestinal epithelial cells during inflammation.^61,62^ This selective use of ethanolamine gives the pathogens a competitive edge over commensal bacteria. As discussed earlier, iron is of critical importance to pathogen fitness in several species of Proteobacteria (e.g., *E. coli*). Iron metabolism is vital for efficient bacterial colonization and presentation in the gut and proliferation in the disseminated bloodstream.^63–66^ During intestinal inflammation, AIEC overexpress the genes encoding propanediol utilization (pdu operon) and iron acquisition (yersiniabactin, chu operon), thereby promoting intestinal inflammation.^37^ Also, it has been shown that enterobactin, a catecholate siderophore secreted by *E. coli*, dampens the activity of myeloperoxidase released from neutrophils in the inflamed gut, thus giving the growth advantage to *E. coli* over other commensals.^67^ Kitamoto *et al*. found that pathogenic *Enterobacteriaceae*, such as AIEC LF82 and *C. rodentium*, shift their metabolism from carbohydrate to amino acid catabolism in the inflamed gut.^68^ In particular, l-serine catabolism is vital for the competitive fitness of these pathogens over commensal bacteria. Intriguingly, l-serine catabolism does not control the fitness of these pathogens in the absence of inflammation, suggesting that l-serine–dependent growth is a selective strategy used by pathogenic *Enterobacteriaceae* during inflammation Figure 2d.

In addition to transcriptional regulation, some bacteria acquire novel functions through horizontal gene transfer (HGT) to adapt better to the surrounding environment. Gut inflammation can boost HGT between pathogenic and commensal bacteria. For example, in *S. enterica* ser. Typhimurium–induced colitis, commensal *E. coli* acquires colicin-plasmid p2 from *S. enterica* ser. Typhimurium, thereby increasing its fitness by evading colicin Ib–mediated killing.^69^ Also, *S. enterica* ser. Typhimurium transfers its prophage SopEΦ to other bacteria in the inflamed gut; thereby enhancing their virulence and fitness.^70^ Moreover, EHEC strains can carry multiple phage-borne *nanS*-p alleles.^71^ As *nanS*-p genes are responsible for the cleavage of mucin *O*-acetyl residues, EHEC that express phage-borne esterases can gain a growth advantage over competing strains by adapting to mucosal niches. Thus, interbacterial HGT (i.e., plasmid, bacteriophage) is a key functional reprogramming mechanism that some pathogens deploy in the inflamed gut.

### 3.3. Commensal bacteria resilience after inflammation

Although the adaptation to the inflammatory environment is a central strategy for pathogenic bacteria to thrive in the gut at the expense of commensal bacteria, the commensal bacteria also harbor similar mechanisms. The avoidance of inflammation-induced growth suppression is often used by commensal bacteria to boost their resilience after inflammation. For example, during inflammation-associated iron limitation, pathogens capture iron using siderophores. Human commensal *B. thetaiotaomicron* does not produce siderophores. However, *B. thetaiotaomicron* uses xenosiderophores – siderophores enterobactin and salmochelin produced by members of the *Enterobacteriaceae* family – for iron acquisition to survive during colitis.^72^ Using RNA-seq analysis, Zhu *et al*. determined that *xusABC* genes are upregulated in *B. thetaiotaomicron* during *Salmonella*-induced gut inflammation. The XusABC system is required for *B. thetaiotaomicron* to capture xenosiderophores produced by enterobacteria.^72^ Indeed, a mutant *B. thetaiotaomicron* lacking the *xusABC* locus is defective for xenosiderophore-mediated iron uptake *in vitro*.^72^ Likewise, commensal bacteria are capable of self-modification to assist colonization during gut inflammation. For instance, commensal Bacteroidetes can modify their lipopolysaccharide structure, resulting in increased resistance to antimicrobial peptides and resilience during gut inflammation.^73^

## 4. Targeting bacterial adaptation strategies to treat disease

Thus far, we have discussed the mechanisms by which commensal and pathogenic bacteria colonize the healthy and the inflamed gut. Notably, pathogenic bacteria use unique metabolic adaptation mechanisms to gain a growth advantage over commensal bacteria or to avoid host antimicrobial immunity. We can target these pathogen-specific strategies to develop potential treatments that selectively suppress the growth of pathogenic bacteria without influencing the beneficial commensal bacteria.

The most straightforward approach is to supply the disease-causing bacteria with metabolic competitors. Various probiotic bacterial strains, such as lactic acid–producing bacteria, have been used to treat bacteria-driven diseases, such as IBD.^74^ Likewise, the transplantation of healthy fecal microorganisms (so-called fecal microbiota transplantation [FMT]) has shown promise in treating *C. difficile* infection and IBD.^75,76^ These bacterial therapies provide metabolically associated bacteria that can directly compete with disease-causing bacteria for nutrient or colonizing niches. In addition, probiotic bacteria and beneficial bacteria contained in healthy microbiota can elicit host protective immunity or anti-inflammatory immunity, which, in turn, helps to combat pathogens and promote the recovery from pathogen-caused inflammatory damage.^77–79^

Targeting the specific inhibitors that impede the metabolic pathways used by disease-causing bacteria is a rational approach to the treatment of disease. Tungstate has been identified as a specific inhibitor of the molybdenum cofactor-dependent microbial respiratory pathways.^80^ This pathway, which is used by pathogenic *Enterobacteriaceae*, such as *E. coli*, only during the period of inflammation, can be inhibited to attenuate colitis and colon cancer caused by pathogenic *Enterobacteriaceae*.^80,81^ Similarly, targeting pathogen-specific nutrients is a selective treatment that prevents colonization by pathogenic bacteria and subsequent disease development. As mentioned, the increase in luminal sialic acids during DSS-induced inflammation promotes the growth of *E. coli*. The oral administration of sialidase inhibitors effectively suppresses the expansion of *E. coli* and subsequently ameliorates DSS-induced colitis in mice.^57^ Likewise, l-serine catabolism promotes the growth of pathogenic *Enterobacteriaceae*, such as AIEC LF82 and *C. rodentium* in the inflamed gut.^68^ Since most of the l-serine available in the gut lumen, and used by the disease-causing pathogens, is supplied by diet, depriving dietary proteins of l-serine can inhibit the expansion of pathogenic *Enterobacteriaceae*, thereby ameliorating the disease they cause.^68^ Thus, inhibition of pathogen-specific metabolic pathways can selectively “starve” the disease-causing bacteria. However, targeting such pathways may not be sufficient to treat the disease caused by those bacteria. As such metabolic pathways are mainly used by pathogenic bacteria to gain a competitive fitness advantage over other resident bacteria,^68^ pathogenic bacteria can survive without these metabolic benefits in the absence of competitors. In this regard, providing suitable competitors (e.g., probiotic treatment, FMT) alongside pathogen metabolic inhibition may more effectively treat the disease caused by those bacteria. Alternatively, supplying diet preferred by competing commensals is an option to promote the indigenous competitors instead of providing competing bacteria exogenously.

In addition to targeting pathogen-specific metabolic pathways, inhibiting pathogen localization may be another option. As discussed earlier, pathogenic bacteria localize in close proximity to the host mucosa to evade nutritional competition with commensals in the gut lumen and to gain access to unique nutrient sources available at the mucosal niche (e.g., host-derived mucus glycans).^82–85^ Therefore, targeting molecules responsible for the colonization of pathogen-specific niches (e.g., mucosa adhesion molecules, flagellar proteins required for mucus penetration, type 3 secretion system) can force pathogens to stand in the same ring with their metabolic competitors.

## 5. Conclusion

The recent advances in next-generation sequencing and mass spectrometry technologies highlight the metabolic landscape in the development of the gut bacterial ecosystem. As discussed, the bacterial metabolic pathways, which are the key mechanisms that regulate the bacterial community, can be targeted for the editing of the gut microbial community. However, it is noteworthy that bacteria can change their metabolic and nutritional preferences according to the surrounding microenvironment, such as inflammation. The bacterial environment-dependent metabolic flexibility, therefore, needs to be considered before editing the microbiota. For example, the deprivation of key nutrients by modulating the diet can efficiently reduce opportunistic growth and infection by pathogenic bacteria in the steady-state gut. However, the same strategy is ineffective once inflammation has developed, as pathogenic bacteria reprogram their metabolic requirements. Thus, the same treatments, namely dietary interventions, may not be equally effective for different patients, or even for the same individuals who are at distinct stages of disease (e.g., grade of inflammation). On the one hand, metabolic flexibility benefits pathogenic bacteria, enabling the avoidance of metabolic limitations. On the other hand, metabolic flexibility ensures the safety of dietary interventions. Certain metabolic pathways only operate in pathogenic bacteria in certain disease conditions (e.g., inflammation); control and regulation of these pathways has no impact on the bacterial ecosystem in the healthy gut. For example, the l-serine–deficient diet does not affect the fitness of pathogenic *E. coli* and *C. rodentium* in the healthy gut, whereas it effectively limits their growth in the inflamed gut.^68^ Thus, this dietary treatment only impacts the gut microbial community during inflammation, not in the steady state. Clearly, the understanding of bacterial metabolic pathways and their flexibility in disease conditions will advance the development of personalized, disease-specific microbiota editing strategies.
